# The global mismatch between equitable carbon dioxide removal liability and capacity

**DOI:** 10.1093/nsr/nwad254

**Published:** 2023-10-01

**Authors:** Pu Yang, Zhifu Mi, Yi-Ming Wei, Steef V Hanssen, Lan-Cui Liu, D’Maris Coffman, Xinlu Sun, Hua Liao, Yun-Fei Yao, Jia-Ning Kang, Peng-Tao Wang, Steven J Davis

**Affiliations:** The Bartlett School of Sustainable Construction, University College London, London WC1E 7HB, UK; Energy and Power Group, University of Oxford, Oxford OX2 0ES, UK; Exeter Sustainable Finance Centre, University of Exeter, Exeter EX4 4PU, UK; The Bartlett School of Sustainable Construction, University College London, London WC1E 7HB, UK; Center for Energy and Environmental Policy Research, Beijing Institute of Technology, Beijing 100081, China; School of Management and Economics, Beijing Institute of Technology, Beijing 100081, China; Department of Environmental Science, Faculty of Science, Radboud University, Nijmegen 6500 GL, The Netherlands; Business School, Beijing Normal University, Beijing 100875, China; The Bartlett School of Sustainable Construction, University College London, London WC1E 7HB, UK; The Bartlett School of Sustainable Construction, University College London, London WC1E 7HB, UK; Center for Energy and Environmental Policy Research, Beijing Institute of Technology, Beijing 100081, China; School of Management and Economics, Beijing Institute of Technology, Beijing 100081, China; Strategy Plan Department, Sinopec Research Institute of Petroleum Engineering, Beijing 100101, China; Center for Energy and Environmental Policy Research, Beijing Institute of Technology, Beijing 100081, China; School of Management and Economics, Beijing Institute of Technology, Beijing 100081, China; Center for Energy and Environmental Policy Research, Beijing Institute of Technology, Beijing 100081, China; School of Management and Economics, Beijing Institute of Technology, Beijing 100081, China; Department of Earth System Science, University of California, Irvine, CA 92697, USA

**Keywords:** carbon dioxide removal, land-based solutions, international equity

## Abstract

Limiting climate change to 1.5°C and achieving net-zero emissions would entail substantial carbon dioxide removal (CDR) from the atmosphere by the mid-century, but how much CDR is needed at country level over time is unclear. The purpose of this paper is to provide a detailed description of when and how much CDR is required at country level in order to achieve 1.5°C and how much CDR countries can carry out domestically. We allocate global CDR pathways among 170 countries according to 6 equity principles and assess these allocations with respect to countries’ biophysical and geophysical capacity to deploy CDR. Allocating global CDR to countries based on these principles suggests that CDR will, on average, represent ∼4% of nations’ total emissions in 2030, rising to ∼17% in 2040. Moreover, equitable allocations of CDR, in many cases, exceed implied land and carbon storage capacities. We estimate ∼15% of countries (25) would have insufficient land to contribute an equitable share of global CDR, and ∼40% of countries (71) would have insufficient geological storage capacity. Unless more diverse CDR technologies are developed, the mismatch between CDR liabilities and land-based CDR capacities will lead to global demand for six GtCO_2_ carbon credits from 2020 to 2050. This demonstrates an imperative demand for international carbon trading of CDR.

## INTRODUCTION

Under the 2015 Paris Agreement, 192 parties (i.e. 191 countries plus the European Union) committed to pursuing efforts to limit the increase of global mean temperatures to 1.5°C [1], which entails reaching global net-zero CO_2_ emissions before the mid-century, and net-zero greenhouse gas (GHG) emissions around 2060 [2]. However, both the net zero targets and the 1.5°C goal require a rapid transition from the current energy system, hard-to-abate sectors and land-use system. Despite the ongoing process of building new power plants, renewable energy sources (e.g. solar, wind and nuclear) will not result in zero life-cycle emissions [3]. Certain hard-to-abate sectors, such as aviation, long-distance transportation and shipping, heavy industry, and construction materials, will continue to produce residual emissions even after reaching net zero [2,4]. While conserving and enhancing land-based carbon sinks will be crucial for generating negative emissions in the future [5], currently, land use is still a source of emissions, contributing 10% of global CO_2_ emissions in 2020 [6]. This is primarily driven by the rising global population and per-capita production of agricultural goods [7].

Given these challenges, all the 1.5°C emissions pathways (as well as most 2°C pathways) in the Intergovernmental Panel on Climate Change’s (IPCC’s) Special Report rely on carbon dioxide removal (CDR) to offset continuing CO_2_ emissions, with the global total averaging 10.5 GtCO_2_ per year in the year net-zero CO_2_ is achieved [8]. A clear demand for CDR exists, but there is a lack of clarity regarding how and when countries should deploy it. This lack of clarity poses a significant obstacle to technology development and the establishment of an international market. Integrated assessment models (IAMs) provide some regional CDR trajectories over time, which incorporate technologies such as afforestation and reforestation, bioenergy with carbon capture and storage (BECCS), and—in a few models like Model for Estimating the Regional and Global Effects of Greenhouse Gas Reductions (MERGE) [9] and REgional Model of Investment and Development (REMIND) [10]—direct air carbon capture and storage (DAC). However, these pathways are mostly at the regional level and do not consider the biophysical and geophysical limits for countries to deploy CDR technologies.

Additionally, despite the importance of CDR in meeting international climate goals, none of the nationally determined contributions (NDCs) quantifies the negative emissions that may be needed to achieve announced targets or identifies which CDR technologies may be deployed within the commitment period [11]. Recent CDR research has focused on evaluating the global potential of different CDR options and their biophysical and economic limits [12–17], but has largely disregarded the profound regional imbalances of CDR in net-zero IAM scenarios: less-developed regions are allocated with more CDR to offset the residual emissions for developed regions [8]. While various allocation plans have been proposed for net emissions [18,19], few studies have addressed how CDR requirements might be shared equitably over time. Some studies have allocated CDR equitably among major economies [20], while others evaluated cumulative removal at the country level [21]. However, it remains unclear how country-level allocations might differ over time and how equitable allocations compare to the biophysical and geological resources available in each country. Understanding these aspects is critical for countries to set their CDR goals for 2030 or the mid-century.

Establishing a separate market for CDR is necessary, as pricing the depletion of the remaining carbon budget differs fundamentally from pricing overshot emissions after the budget has been depleted [22,23]. As a prerequisite for establishing an international negative-emission trading market, national CDR liability allocation needs to be estimated over time and compared with domestic CDR capacity [24]. Moreover, it is important to stress that CDR cannot replace emission reductions [25], and thus, equitable CDR deployment should be presented in conjunction with mitigation efforts.

Here, we allocate global CDR requirements from 13 1.5°C IAM scenarios [26] among 170 (of 191) countries that signed the Paris Agreement. Due to the lack of consensus around equity principles, we base our discussion on three fundamental dimensions of equity: countries’ ability to pay for CDR (capability [27,28]), population (equality [29,30]) and historical emissions (responsibility [31,32]). Socioeconomic factors heavily influence both the global demand for CDR and country-level allocations [18,33]. We assess uncertainties in CDR allocations by analysing the 13 IAM scenarios under their corresponding shared socioeconomic pathways (SSPs). As CDR is meant to complement efforts in hard-to-abate sectors rather than replace mitigation, we also present equitable CDR as the CDR ratio to residual emissions (i.e. the emissions from hard-to-abate sectors) to better track CDR’s alignment with 1.5°C-consistent emissions reduction, thus guiding the development of NDCs. Moreover, considering each country's biophysical endowments to deploy CDR and geophysical endowments to permanently store carbon, we compare equitable CDR allocation with cost-effective land-based removal potential [5], BECCS potential and storage potential [34]. The study examines 10 different CDR options (detailed documentation of the technologies can be found in the Supplementary Data and literature [5,35]) for land-based CDR endowment. The cost-effective potential is estimated under a carbon price of US$100/tCO_2_, while the benchmark is determined by the midpoint of the carbon price range in 2030 from cost-effective 1.5°C IAM scenarios [5,36].

## RESULTS AND DISCUSSION

### Substantial mitigation is needed along with CDR

**Figure 1.**
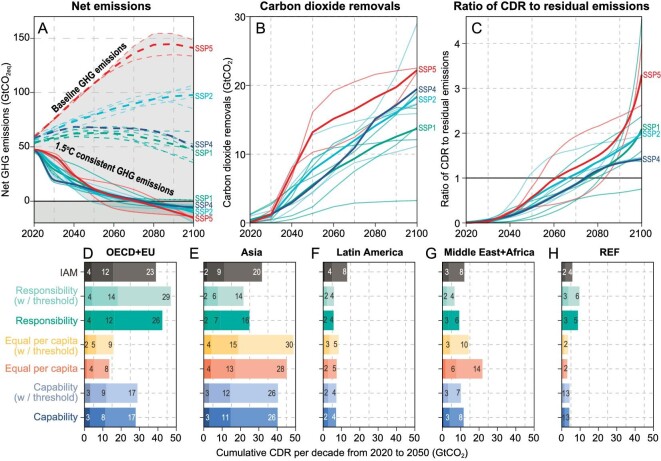
Global CDR pathway for allocation, the ratio of CDR to residual emission, and a comparison between IAM results and equitable allocations. (A) Net GHG emissions. Baseline trajectories are shown in dashed lines, and 1.5°C-consistent pathways are represented by solid lines. Emission pathways are sourced from the IPCC's SR1.5 database. (B) Global CDR pathways for allocation. (C) The ratio of CDR to residual emissions. Society achieves net zero when the ratio equals one. (D–H) Regional CDR per decade (2020–2030, 2030–2040, 2040–2050) from IAM and equitable allocation principles.

Emissions pathways aligned with the 1.5°C goal (Fig. 1A, selected SSP scenarios from IPCC SR1.5 [26]) require both current emission mitigation and negative emissions from CDR (Fig. 1B). In 2050, the average mitigation (60.1 GtCO_2_) of 13 scenarios is approximately 8 times the amount of CDR (7.4 GtCO_2_), highlighting the significant mitigation efforts needed alongside CDR to achieve the 1.5°C target. While baseline emissions may vary across SSPs, net emissions consistent with the 1.5°C goal remain relatively consistent (Fig. 1A, based on [26]). Low GHG emissions in scenarios like SSP1 enable a smoother low-carbon transition by the end of the century, while scenarios with high baseline emissions (e.g. SSP5) require both greater emissions reductions and CDR. No feasible 1.5°C scenarios are observed under SSP3 due to intense regional rivalry and countries’ focus on energy security.

To contextualize the potential demand for CDR, we calculate the ratio of CDR as share of residual GHG emissions (Fig. 1C). In many scenarios, the amount of CDR required exceeds residual GHG emissions, with the ratio gradually increasing from 0 (where no CDR is available) to 1 (where all emissions can be offset by CDR, achieving net zero), and higher when CDR removes more GHGs than are emitted. The average ratio of CDR to residual GHG emissions from the 13 selected scenarios (selected if they are modelled under SSPs; see Methods) is 0.37 in 2030, rising to 0.44 in 2050 and 0.73 in 2060.

Analysing the cumulative CDR per decade from 2020 to 2050 (Fig. 1D–H), cost-effective IAMs predominantly assign CDR liability to OECD + EU (Organisation for Economic Co-operation and Development, and European Union) and Asian countries, where carbon removal costs are expected to be lower. The IAMs allocate CDR based on global cost-effectiveness, which suggests Latin American countries conduct 1.1 GtCO_2_ CDR during 2020–2030 while substantially increasing the number to 3.8 GtCO_2_ during 2030–2040 and 8.1 GtCO_2_ during 2040–2050. The cost-effective CDR for Latin America amounts to 13.1 GtCO_2_ during the 2020–2050 period, higher than all the equitable allocation suggests (ranging from 5.7 GtCO_2_ to 8.4 GtCO_2_). For other regions, the equitable allocation usually means a higher CDR deployment compared to IAM scenarios. Allocation based on responsibility suggests that the OECD + EU countries and Reforming Economics do more than the cost-effective level due to their high historical emissions. Allocation based on capacity and equity assigns greater CDR liability to Asia and the Middle East + African countries, considering their higher population share and projected rising GDP per capita during 2020–2050. If individuals were to equally share CDR responsibility, these two regions would need to conduct more CDR than suggested by the IAMs.

What becomes clear from these scenarios is that a large gap exists between the 1.5°C-consistent CDR demand and current levels of CDR deployment. The six IAMs whose outcomes we analyse model CDR as either afforestation/reforestation or BECCS. The latter is one of the most mature CDR technologies and may to some extent be considered a proxy for a wider range of CDR options, as the prevalence of BECCS in IAMs may decrease when other CDR options are included [14]. Average CDR deployment across the analysed scenarios is 291 (0–1266) MtCO_2_ in 2020, of which 31 (0–103) MtCO_2_ is from BECCS deployment, with the remaining carbon sequestration resulting from the land-use sector (afforestation/reforestation). This becomes 714 (6–1774) MtCO_2_ in 2030. For comparison, in 2019 there were 11 operational BECCS facilities recorded [37]. Only five facilities actively use BECCS technologies, collectively capturing ∼1.5 MtCO_2_/year [38]. The most recent estimate for 2023 suggests a global BECCS capacity of 1.82 MtCO_2_/year [39]. Based upon an average between 2019 and 2023, 1.5°C scenarios already model 19 times more BECCS in 2020 than those currently in place, displaying the current gap between modelled and actual deployment.

### Allocating CDR liability to the world's 10 major economies

**Figure 2.**
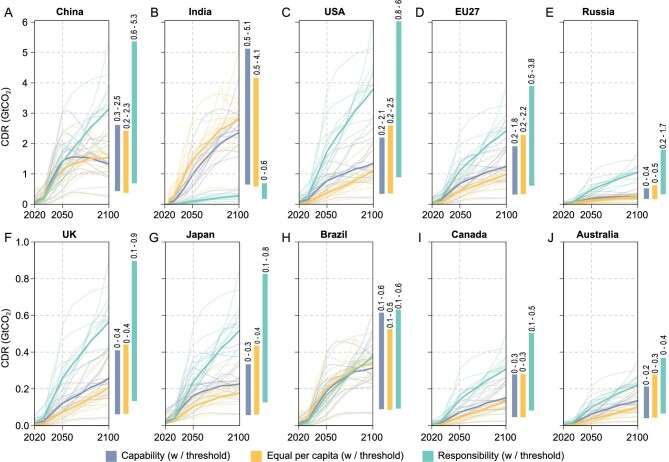
Equitable CDR pathways from 2020 to 2100 for 10 economies. (A–J) Allocations adopt three equity principles with a participation threshold, and only allocate CDR liability to countries with a GDP per capita higher than the threshold. Capability allocates CDR quotas based on GDP. Equal per capita allocates quotas based on population. Responsibility allocates quotas based on historical cumulative emissions. The error bar shows the range of CDR in 2100 from 13 IAM scenarios.

Figure 2 shows carbon removal allocated to 10 countries/regions using three equity principles with participation threshold (results for 170 countries and 6 principles are provided in the Supplementary Data). The 10 countries/regions include 7 of the largest developed economies, namely, the USA, the EU, Russia, the UK, Japan, Canada and Australia, and 3 representative developing countries from the BASIC group, namely, Brazil, India and China. The following analysis compares the average of 3 equitable allocation results under 13 IAMs to compare parties’ liability in general.

Allocation of the global CDR to countries is comparatively high in the near future. Taking the average of the 3 equity principles, CDR targets for China, the USA and the EU are 108 MtCO_2_, 80 MtCO_2_ and 67 MtCO_2_ in 2022, respectively, while the removal of another 79 MtCO_2_ is allocated to the other 7 parties (Fig. 2). In 2030, this removal liability would triple for most countries, resulting in 237 MtCO_2_ for China, 129 MtCO_2_ for the USA and 136 MtCO_2_ for the EU. India will follow with a target that has increased 8-fold to 129 MtCO_2_ due to its rising capability to deploy CDR and its increased responsibility. But if India follows the 1.5°C-consistent emission trajectory, their CDR liability will stay at a low level no higher than 0.6 GtCO_2_. The other 6 parties share a total of 164 MtCO_2_ CDR liability in 2030.

At present, all 10 parties have announced their intention to achieve net-zero targets around the 2050s, sending a clear signal that CDR technology should be enhanced [40]. CDR development has, however, thus far been limited in scale and geographically unevenly distributed. Of the 11 operational BECCS facilities reviewed by the Global CCS Institutes in 2019, 6 were in the USA [38]. Norway's Full Chain CCS is also planning to operate BECCS, with a maximum potential capacity to capture 0.8 MtCO_2_/year, and several small demonstration and pilot BECCS projects are under construction in Canada, Japan, England and France [38]. As of 2021, there are 16 DACCS projects currently operating in Canada, Europe, Iceland and the USA [37]. In September 2021, the DACCS project in Iceland (named Orca) started operating; it has the capacity to remove 0.4 MtCO_2_/year [41]. The Orca project cooperates with Carbfix and buries the collected CO_2_ as rocks deep underground, which provide stable storage for millennia; this is considered to be one of the few negative emission projects [42]. Afforestation and reforestation have been recognized and deployed in recent decades, yet the verification of removal is still challenging and needs to be provided case by case. In general, progress is still far from being aligned with 1.5°C-consistent pathways.

### Implications of different equitable CDR allocation principles for 10 major economies

Different equity principles lead to substantially different allocated CDR shares (Fig. 2). Countries with a high GDP show the capability to deploy CDR technology. Therefore, most of the selected parties shared a large proportion of CDR quotas under the capability principle. With rapid projected economic development, the allocated CDR liabilities of China, India and Brazil swiftly increase. The equal per-capita approach, also known as the equality principle, argues that every individual should have the same right, but applied in this context, also the same CDR burden. Applying the equality principle to allocate CDR quotas would add burdens to developing countries with a large population size (e.g. China and India) while lifting the liabilities of developed countries. The responsibility principle allocates global CDR quotas based on cumulative national emissions since 1850, providing the highest CDR quota for most selected parties. The historical emissions of emerging economies (i.e. China, India and Brazil) are relatively low at the beginning. Nevertheless, with high emission projections under both SSP scenarios, their CDR liabilities also increase rapidly. The additional participation threshold exempts the removal liability of countries with a low per-capita GDP, therefore increasing the CDR quota of developed economies. However, such an increment would not be significant until the 2050s, when global CDR requirements in the pathways increase.

### CDR liability as a ratio to residual emissions

**Figure 3.**
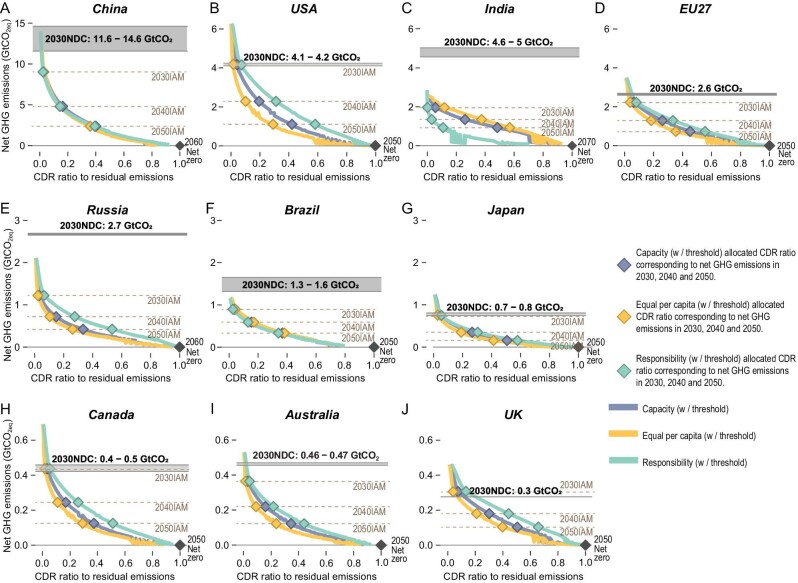
Net GHG emissions and corresponding ratio of CDR to residual emissions for 10 countries. (A–J) The grey solid lines indicate the (range of) net GHG emissions committed in national NDCs for 2030. The grey dashed lines represent the average net GHG emissions modelled for countries by IAMs for 2030, 2040 and 2050. Coloured diamonds denote the CDR to residual emission ratio under three equity principles and the corresponding net GHG emissions from IAMs for 2030, 2040 and 2050.

As carbon removal would be required in addition to decarbonization, CDR requirements should be seen in the context of GHG emission reduction. This section further discusses the ratio of CDR to residual GHG emissions for the 10 major economies, by presenting the relative CDR liability per national net GHG emissions (Fig. 3). The CDR ratio to residual emissions reflects how much removal is needed relative to total GHG emissions emitted to the atmosphere (net GHG emissions plus CDR). Given that NDCs and net-zero commitments are based on net GHG emissions rather than total emissions, we compare the relative CDR ratio with net GHG emissions and further mark the range of NDCs and net-zero commitments. Note that the net GHG emissions here are taken from IAM scenario outcomes, assuming countries will reduce their positive emissions and deploy CDR in a global cost-effective approach [26] (net GHG emissions are provided in Supplementary Data). The global cost-effectiveness is ensured by a strong market governance of international mitigation/removal trading mechanisms (e.g. the Clean Development Mechanism (CDM)), which transfer the mitigation effort from developed countries with high abatement costs to developing countries with low abatement costs, thus equating marginal abatement costs among all countries [43].

As shown in Fig. 3, a sharp increase in the CDR ratio of residual emissions is needed to achieve the net-zero GHG target for all countries. Given national NDC commitments for 2030 and net-zero GHG commitments in the mid-century, a rapid energy transition is required to lower emissions [44]. In 2030, the three equitable CDR allocations suggest an average of 0.05 (ranging from 0.003 to 0.13) CDR in residual GHG emissions for the 10 major economies, while this ratio is ∼0.03 (ranging from 0 to 0.36) when averaged across all 170 countries that are studied here. By 2025, China's net GHG emissions are projected to be 12 GtCO_2_, while 156 MtCO_2_ (≈0.01 of residual GHG emissions) should be offset according to equitable allocation. In these estimations, net GHG emissions in the USA and EU are 5.4 GtCO_2_ and 2.9 GtCO_2_, respectively, while their CDR should account for 0.02 and 0.03 of their total GHG emissions. The CDR ratio is the highest in the UK, accounting for 0.04 of its total emissions in 2025 and 0.08 in 2030. Using the equitable allocation principle with participation threshold, India does not join the CDR allocation until 2024–2027 (with a slight difference under alternative SSPs) given its low GDP per capita. However, using the equality principle, India's CDR ratio becomes 0.04 of its total GHG emissions in 2025 (the average ratio across three equity principles is 0.02) and rises to 0.12 in 2030 (the average ratio across three equity principles is 0.06), according to its high population.

The fact that the current national commitment in 2030 (according to the post-2020 updated NDCs) is much lower than required in a 1.5°C-consistent mitigation pathway (marked as ‘2030 IAM’ in Fig. 3) makes it clear that emission reduction is currently insufficient, which means further emission reduction along with CDR is required (Fig. 3). The NDCs of India and Russia are much higher than the IAMs suggest, indicating some countries with higher abatement costs can transfer their mitigation effort to these countries and lower global emissions in a cost-effective way. Note that the above CDR allocation is only 1.5°C-consistent with a substantial amount of mitigation and assuming that global GHG emissions are in line with IAM 1.5°C pathways. However, the updated NDC target at 2030 totals 55.2 GtCO_2eq_ for 170 countries (ranging from 51.9 to 58.5 GtCO_2eq_) [44], while 13 1.5°C scenarios suggest only 33.5 GtCO_2eq_ (ranging from 18.8 to 49.2 GtCO_2eq_) GHG emissions. The result implies that a further 21.7 GtCO_2eq_ emission reduction is needed, either by conducting more mitigation or promoting more ambitious CDR, to make the above allocation consistent with 1.5°C.

### Domestic capacity for CDR deployment

The ability to deliver CDR domestically depends on the distribution of biophysical capacity and the availability of suitable geological sites for CO_2_ storage. It is essential to compare national CDR liability with the capacity to address global inequality in CDR. However, there is significant variation in equitable allocation results, which has led to a lack of consensus on equity principles. To account for this diversity while maintaining conciseness in subsequent expressions, we calculate the average equitable allocation of CDR based on three basic equity dimensions. This section presents a comparison of the average equitable CDR allocation with the potential for land-based CDR and geological storage capacity, costing less than $100/tCO_2_. This comparison highlights the potential gaps between a country's CDR liability and its capacity to deliver CDR. Identifying these gaps can help policy makers make informed decisions to address global CDR inequality.

**Figure 4.**
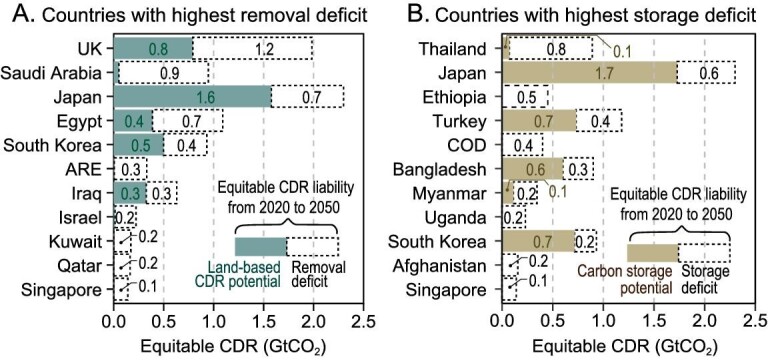
The gap between cumulative national CDR capacity and CDR liability for 2020–2050. (A) Top 11 countries with less land-based CDR potential. (B) Top 11 countries with less geophysical storage potential. COD: the Democratic Republic of the Congo. ARE: United Arab Emirates.

Twenty-five countries have a lower cost-effective land-based CDR potential than the allocated equitable CDR quotas derived in this study, with a shortage of 5.9 GtCO_2_ in total (Fig. 4A). On the other hand, countries such as Brazil, Indonesia, the USA and Russia theoretically have the capacity to meet their CDR liabilities with theoretical additional land-based removal capacities of 20.9, 15.0, 9.6 and 9.0 GtCO_2_, respectively. The UK faces the highest land-based removal capacity shortage, with a gap of 1.2 GtCO_2_ needing to be filled by technical (non-land-based) CDR options if the UK is left without an international removal transfer mechanism. Saudi Arabia, Japan and Egypt are other examples of countries with a shortage of removal capacity, with gaps of 0.9, 0.7 and 0.7 GtCO_2_, respectively.

Only 84 countries have cost-effective and accessible geophysical storage, which makes the mismatch between storage capacities and CDR liabilities even more severe. For the purpose of this matching analysis, we consider the EU-27 as a single region. We then find that 71 out of 170 countries have geographical carbon storage capacities that are lower than their removal liabilities, while the countries with the highest storage deficits are shown in Fig. 4B. The result reveals a notable disparity in carbon storage capacity, where the total storage shortage adds up to 6.4 GtCO_2_. But countries such as Russia, Australia and Brazil could cover this with their potential extra storage capacity of 247, 196 and 171 GtCO_2_, respectively.

There are 12 countries that have both lower land-based CDR potential and lower carbon storage potential compared to their allocated CDR share. Japan, South Korea and Singapore are those with considerable CDR liability but lack CDR capacity (>0.1 GtCO_2_). These countries can either purchase the certified emission reduction credits or develop more advanced CDR that can permanently store carbon in the deep sea or does not require carbon storage, such as the mineralization of CO_2_ in basalt (as applied in Carbfix), to reduce this mismatch situation [45,46]. As shown in Fig. 4, a lack of CDR capacity is usually associated with small or low-income countries. To add a perspective to the open discussion of effort sharing, we propose a potential-based CDR allocation in Supplementary Data, which allocates CDR quotas based on countries’ land-based CDR potential.

After exhausting all land-based carbon storage and geophysical carbon storage potentials, eight countries do not have the capacity to meet their liability domestically if they aim to meet the average of the six equitable CDR allocations. Among them, Singapore faces the highest shortage at 140 MtCO_2_, followed by Lebanon at 59 MtCO_2_. In order to meet these shortages, offset credits must be purchased overseas, creating potential economic opportunities for countries with high CDR potential. There are currently 95 MtCO_2_ of offset credits retired (cannot be transferred or used) domestically and internationally in 2021 [47]. However, the lack of CDR in countries such as Singapore and Lebanon will generate an international market that is twice as large as the current market.

### Challenges to realizing the full potential of CDR

For CDR to realize its full potential, several prerequisites must be met. These include supportive institutional, social and economic conditions, land availability, water availability, favourable carbon prices and zero or minimal trade-offs with other environmental protection goals, including biodiversity conservation. Institutional, social and economic conditions are preliminary, as land-based removal requires professionals with expertise in engineering science, agriculture and forestry. The country would need to invest in education and training programmes. In addition, equitable CDR allocations can only be considered fair after food production is protected. The potential estimates consider land competition among CDR technologies but not water and food competition. When food and water are prioritized, the CDR potential will be lower than expected, as technologies such as BECCS may compete for land and water with crop production or at least drive up food prices. Furthermore, in our potential analysis, it is assumed that all geophysical storage is exclusively allocated towards carbon removal and does not compete with carbon capture and storage from fossil fuels. Additionally, it is assumed that countries have access to all the other necessary inputs, such as biomass feedstocks for BECCS or clean energy for DACCS. When taking these additional considerations into account, the gaps in CDR capacity may potentially be even larger.

Research has demonstrated that delaying action in the deployment of CDR technologies can lead to a reduction in biophysical potential and an increased reliance on CDR due to the cumulative effects of CO_2_ [21,48]. If a 10-year delay were to occur, resulting in a 15% decrease in national land-based CDR potential, a 5% decrease in storage potential and a 10% increase in equitable CDR to offset and compensate for the cumulative effects of additional emissions due to the delay [21], an additional 36 countries (including 27 EU countries) may face insufficient domestic CDR capacity to meet their liabilities, increasing the gap between liability and capacity from 5.9 GtCO_2_ to 14.6 GtCO_2_. These findings underscore the potential consequences of inaction and highlight the urgent need to quickly bring about emission reduction and start the deployment of CDR technologies to avoid exacerbating global CDR inequality.

Given the global mismatch between CDR liability, equity and capacity, there is potential benefit to be gained by countries considering the formation of a supra-national bloc (climate clubbing) to share CDR capacity. For example, analysis of national liability and land-based CDR capacity in the EU reveals that out of the 27 EU countries, 11 currently lack the domestic capacity to meet their liability, resulting in a deficit of 2.7 GtCO_2_. However, if these countries come together as a bloc, they can pool their resources and potentially even achieve a surplus capacity of 0.2 GtCO_2_. Land-based potential in our analysis does not include BECCS, as afforestation and reforestation will compete with it for available land. However, for the IAM-based mitigation pathways, BECCS is used as the main CDR technology, as discussed above, and could be considered, to some extent, as a proxy for other CDR technologies [14]. BECCS potential below $100/tCO_2_ is, however, only 7% compared to the overall land-based removal potential at these costs, and BECCS’s potential (below 100$/tCO_2_) alone is far less than the allocated CDR liability for all countries (Supplementary Fig. 4). This highlights the importance of advancing other CDR technologies. Furthermore, to allow further feasibility assessment and economic analysis of land-based and other CDR approaches, they should be integrated into these modelling tools. Note that the above land-based removal potential is estimated with a carbon price of $100/tCO_2_eq, which means that the potential removal capacity can also be lower if the carbon price is not as high.

If the carbon price rises to the point where it is higher than $100/tCO_2_ and if it were to be implemented globally, global CDR potential would increase, but the inequality in capacity would persist. In our calculations, land-based CDR potential for 2020 to 2050 could rise by 137% if the carbon price constraint is removed. However, only six countries (i.e. Comoros, Iraq, Israel, Lebanon, Oman and the United Kingdom) can fulfil their liability with increased land-based CDR. Both comparisons clearly show that the land-based removal potential is limited, domestic removal may be difficult to achieve, and meeting equitable removal responsibilities would require multiple removal options from both land-based and technical approaches, as well as an international CDR trading mechanism.

## CONCLUSIONS

Our analysis allocates the CDR requirements of 1.5°C pathways to countries based on six different equity principles and compares these allocations with countries’ cost-effective land-based removal and geophysical storage potentials. These country-level results provide tools to track national CDR progress and underscore the need for increasing CDR development, including both land-based and technological CDR, as well as facilitate the development of international trade in CDR obligations.

Large-scale CDR deployment without corresponding efforts to reduce emissions may challenge the feasibility of the Paris Agreement goal [49]. Unlike some mitigation options that come with environmental and health co-benefits, CDR adoption can bring limited direct benefits to the local community [25], and is even associated with multiple risks to land, water, energy and nutrient availability [50], as well as potential impacts on biodiversity [51], depending upon the type of CDR. To target CDR along with emission reduction, we propose an indicator reflecting the ratio of CDR to residual emissions. Assuming that countries’ emissions follow 1.5°C pathways, the average removals allocated according to the six equity principles account for 4% of continuing (residual) GHG emissions in 2030 and 17% in 2040. With increasing deployment of CDR and decreasing emissions, the ratio between the two will be a useful indicator of the national climate effort on both mitigation and CDR.

At the country level, carbon storage potential and land-based CDR potential, including BECCS, are often smaller than national CDR liabilities. Given this unequal distribution of national biophysical and geophysical endowments and the equitable national CDR liabilities identified in this work, development of an international trading system for negative carbon credits is imperative and urgently needed [52]. A market specifically targeting carbon removal obligations is needed for residual emissions offsets [22], while our results show the potential market size from 2020 to 2050. Without significant technological improvement, a total amount of 5.9 GtCO_2_ is needed for countries lacking land-based CDR capacity. The opportunity exists for Latin American countries and South African countries, which have abundant CDR potential after fulfilling domestic liabilities. Providing tradeable and verifiable carbon credits can help these countries’ governments increase their financial capacity for adaptation and mitigation. Countries can also encourage private companies to participate in verifiable CDR supply in addition to national CDR targets, as companies are increasingly pledging to achieve net-zero GHG emissions by the 2050s.

While the results reveal a discrepancy in CDR capacity, stakeholders can proactively secure high-quality carbon offsets early on to achieve their net-zero goals. Firms can strategically allocate resources and investments to these countries with significant CDR potential. By incorporating these findings, stakeholders can enhance their carbon neutrality efforts and contribute to a more sustainable future. There is an urgent demand to scale up CDR capacity in time to keep 1.5°C targets within reach, but the demand for CDR is still ambiguous in national climate commitments. Although the principles of distributing CDR can be an open discussion, it is critical to allocate global CDR requirements to different countries and over time to allow for verifiable targets. Intertemporal instruments to guide CDR deployment at the national level may also send a positive signal to investors and motivate further innovations. Given the level of CDR implied by our analysis, international cooperation and international CDR trading are imperative. The quicker we act, the better our chances are of reaching equitable CDR.

## METHODS

### Scenario description

We use 6 equity principles to allocate global CDR quotas to 170 countries from 2020 to 2100. The CDR quotas are derived from 14 1.5°C-consistent scenarios in the IPCC SR1.5 database [26] (https://data.ene.iiasa.ac.at/iamc-1.5c-explorer/#/workspaces). Socioeconomic factors, such as economic output and demographic characteristics, are critical determinants of national emissions and CDR capacity for meeting the 1.5°C goal. Meanwhile, GDP and population also serve as indicators of capability and equity-per-capita principles. Therefore, we include only the scenarios estimated under SSPs to eliminate inconsistency.

Historical data since 1850 are used to characterize responsibility. Future projections of GDP, population and GHG emissions follow the SSPs. The SSPs provide five alternative GDP and population pathways until 2100, while the six IAMs estimate future GHG emissions under these socioeconomic assumptions. However, the model results are mostly given in 10-year intervals, and models may group the countries differently. Therefore, we adopt country-resolved SSP data, which downscale the regional GDP, population and GHG emissions to countries [53]. The data set provides harmonized socioeconomic data and emission data from 1850 to 2100, and the 10-year data are interpolated as annual data. We choose the convergence data among the three downscaling methods, assuming exponential convergence of emissions intensities and convergence before the transition to negative emissions.

### Equity principles: responsibility, capability and equality

In 1992, the parties to the United Nations Framework Convention on Climate Change (UNFCCC) enshrined the concept of ‘common but differentiated responsibility and respective capabilities’ as Principle 7 of the Rio Declaration. Several equity principles were designed to operationalize such concepts and allocate the global carbon budget to countries. The IPCC Fifth Assessment Report reviewed all these equity principles, and three criteria have been identified as the foundation for all allocations, namely: responsibly, capability and equality [19,54–56].

The responsibility approach allocates global CDR quotas by historical emissions [31]. The principle establishes that countries with high emissions in the past are responsible for the current climate damage. Therefore, countries with high cumulative historical emissions should take a higher CDR quota in the future. The equation is as follows:


(1) \begin{eqnarray*}
CCDR\_RE{S}_{s,i,t} = \frac{{\sum\nolimits_{1850}^t {{E}_{s,i,t}} }}{{\sum\nolimits_{i = 0}^{170} {\sum\nolimits_{1850}^t {{E}_{s,i,t}} } }}*GCD{R}_{s,t}.
\end{eqnarray*}

Under one of the five SSP scenarios, *s*, the national CDR quota for country *i* in year *t* is denoted by $CCDR\_RE{S}_{s,i,t}$. Responsibility has been accounted for from 1850 onwards, and the national CDR quota in year *t* is related to its cumulative historical emission share. Emissions are totalled each year based on the 14 scenarios under the SSPs. In other words, the more countries emit each year, the higher their responsibility to remove carbon in the future. The total CDR needed in year *t* under scenario *s* is denoted by $GCD{R}_{s,t}$.

The capability principle addresses the ability to remove carbon and allocate global CDR quotas by the national share of GDP each year [27]. It assumes that countries with a higher GDP will have a greater capability to deploy CDR technology and should bear a higher quota to remove carbon. The equation is as follows:


(2) \begin{eqnarray*}
CCDR\_CA{P}_{s,i,t} = \frac{{GD{P}_{s,i,t}}}{{\mathop \sum \nolimits_{i = 1}^{170} GD{P}_{s,i,t}}} * GCD{R}_{s,t}.
\end{eqnarray*}

Under scenario *s*, the national CDR quota for country *i* in year *t* is denoted by $CCDR\_CA{P}_{s,i,t}$. The country's GDP is denoted by $GD{P}_{s,i,t}$, which follows different SSP projections. National CDR is proportional to the global CDR quota $GCD{R}_{s,i,t}$.

Following the equality principle, every individual makes an effort to remove carbon, and global removal demands are allocated to countries based on their population [57]. The equation is as follows:


(3) \begin{eqnarray*}
CCDR\_EQ{T}_{s,i,t} = \frac{{PO{P}_{s,i,t}}}{{\mathop \sum \nolimits_{i = 1}^{170} PO{P}_{s,i,t}}} * GCD{R}_{s,t}.
\end{eqnarray*}

Under scenario *s*, the national CDR quota for country *i* in year *t* is denoted by $CCDR\_EQ{T}_{s,i,t}$. The country's population is denoted by $PO{P}_{s,i,t}$, which follows different SSP projections. Countries with a larger population will take a larger share of the global removal $GCD{R}_{s,i,t}$.

### Participation threshold

The deployment of CDR technology needs countries to have a certain level of economic development. We follow previous research and set a participation threshold for countries that depend on their GDP per capita [58]. Countries with a GDP per capita higher than the threshold participate in CDR allocation, while low-income countries do not share removal responsibility. The threshold was set at 45% of Annex I countries’ GDP per capita in 1990 [59]. With this participation threshold, the allocation equation above is changed as follows:


(4) \begin{eqnarray*}
CCDR\_RES\_T{R}_{s,i,t} &=& \frac{{\mathop \sum \nolimits_{1850}^t {E}_{s,i,t}}}{{\mathop \sum \nolimits_{i = 1}^{170} \mathop \sum \nolimits_{1850}^t {E}_{s,i,t}*T{R}_{s,i,t}}}\\
&& *\, GCD{R}_{s,t}*T{R}_{s,i,t},
\end{eqnarray*}

(5) \begin{eqnarray*}
CCDR\_CAP\_T{R}_{s,i,t} &=& \frac{{GD{P}_{s,i,t}}}{{\mathop \sum \nolimits_{i = 1}^{170} GD{P}_{s,i,t}*T{R}_{s,i,t}}}\\
&& *\, GCD{R}_{s,t}*T{R}_{s,i,t},
\end{eqnarray*}

(6) \begin{eqnarray*}
CCDR\_EQT\_T{R}_{s,i,t} &=& \frac{{PO{P}_{s,i,t}}}{{\mathop \sum \nolimits_{i = 1}^{170} PO{P}_{s,i,t*}T{R}_{s,i,t}}}\\
&& *\, GCD{R}_{s,t} *T{R}_{s,i,t},
\end{eqnarray*}

where $T{R}_{s,i,t}$ is a binary variable. $T{R}_{s,i,t}$ = 1 when country *i*’s GDP per capita is higher than the threshold in year *t*; $T{R}_{s,i,t}$ = 0 when country *i*’s GDP per capita is lower than the threshold in year *t*.

### Comparison with land-based removal, BECCS and carbon storage potentials

The domestic capacity to deliver CDR with natural resources is ultimately dictated by the distribution of the capacity to remove carbon and store captured carbon dioxide in geological sites. To assess the feasibility of countries’ implementation of CDR technology, we compare national removal liabilities (equitable CDR quotas) with countries’ biophysical endowment to remove carbon and their geophysical endowment to store carbon. Both the CDR removal potential and carbon storage potential are estimated with a carbon price of $100/tCO_2_.

In the IPCC Sixth Annual Report (Working Group I Chapter 5), CDR options are divided into four categories: enhanced biological production and storage on land, enhanced biological production and storage in coastal areas and in the open ocean, enhanced geochemical processes on land and in the ocean, and chemical approaches [60]. All of these approaches have different limitations and challenges, whether related to scalability, permanence, cost, the impact on land-use change and/or biodiversity, or other aspects. If poorly planned or implemented, these approaches can also entail potential risks to food security, biodiversity, and water quality and quantity [5,61]. The land-based removal approach has larger removal potentials and is tied up with public and private policies [62]. Land-based mitigation efforts account for roughly a quarter of the total emission reductions planned in countries’ NDCs [63]. This analysis takes the latest estimation of land-based mitigation potentials from Roe *et al.* [5], which includes the following 10 negative emission options within the following 4 categories from 2020 to 2050: forest and other ecosystem management (including improved forest management; grassland fire management), forest and other ecosystem restoration (including afforestation and reforestation; mangrove restoration; peatland restoration), carbon sequestration from agriculture (including agroforestry, biochar from crop residues, soil organic carbon in croplands, soil organic carbon in grasslands), and BECCS. As BECCS will ultimately store the carbon underground, and because the significant amount of land required for energy crops would not be available for other land-based CDR (see Hanssen *et al.* [13]) and thus cause double-counting with afforestation and reforestation, the overall land-based potential excludes BECCS here, and BECCS potential is separately shown. Since the technical potential may not be feasible due to economic and social constraints, we adopt only their cost-effective potential estimates (with carbon price <$100/tCO_2_) for comparison.

The carbon storage potential calculation is based on Wei *et al.* [34], but further considers the cost-effective constraint (with carbon price <$100/tCO_2_). The potential estimates include geological sites in deep saline formations and oil/gas basins that can be matched with a carbon source from a cost-effective perspective. The carbon storage potential of 794 basins distributed in 84 countries around the world and suitable for carbon storage has been estimated. All sinks can be matched with carbon sources in a cost-effective way (minimum total investment required). Due to technical difficulties, the carbon storage potential for EU 27 countries is estimated and compared as a whole.

## Supplementary Material

nwad254_Supplemental_Files
