# Supplementary material for: The global mismatch between equitable carbon dioxide removal liability and capacity
Source: Natl Sci Rev. 2023 Oct 1;10(12):nwad254. doi: 10.1093/nsr/nwad254 (PMC10659237; doi:10.1093/nsr/nwad254)
Supplement: nwad254_Supplemental_Files [file nwad254_supplemental_files.zip › Supplementary information_Yang et al..docx]

Supplementary information

# 1 Technological portfolio of land-based CDR

## Definition and characteristics

This section outlines the land-based Carbon Dioxide Removal (CDR) technologies included in our potential estimates. While research discussions often focus on technology-based CDR options like BECCS (Bioenergy with Carbon Capture and Storage) and DACCS (Direct Air Carbon Capture and Storage), non-BECCS land-based CDR options are generally more technologically mature (Table S1). Feasibility for CDR can be presented by two aspects, technology readiness level (TRL), and the viability to monitor, report, and verify (MRV) the carbon removal. In this section, we show for ten land-based CDR technologies evaluated by Roe et al.[1]. The technology's TRL, cost at scale, mitigation potential range (without a <$100/tCO2 carbon price constraint), and feasibility of monitoring, reporting, and verification (MRV) are also shown based on the recently published 'State of Carbon dioxide removal' report [2] (Supplementary Table 1).

For the regional and country-level CDR potential estimates, we rely on the estimation provided by Roe et al.[1] because of its cost-benefit perspective, which incorporates a $100/tCO2 constraint. This constraint leads to lower estimations compared to those without a price constraint[3]. While Roe et al.[1] examines 20 land-based mitigation options, we focus on 9 of them for the land-based CDR potential estimates that result in net negative emissions. Although BECCS estimates are discussed, they are not included in the land-based CDR potential as they may conflict with Afforestation and Reforestation efforts.

The TRLs used in this assessment range from 1 to 9, with 1 representing technologies based on observed basic principles and 9 indicating operationally proven systems. These TRL estimates are adopted from the 2023 state of CDR report[2]. MRV is also presented, scoring the simplicity and

Supplementary Table 1 Definition of the land-based CDR technologies (integrating information from [1] and [2])

| Category | Technology | Definition*  (*Where two definitions are given, two separate original studies were considered by Roe et al [1], here we present averaged information across the two. ) | | TRL | Cost at scale | Mitigation potential (GtCO_2_/yr) | Monitoring, Reporting and Verification |
| --- | --- | --- | --- | --- | --- | --- | --- |
| Forests and other ecosystems-management | Improved forest management | Avoided emissions and enhanced sequestration from improved natural forest management, including reduced-impact logging, extended harvest rotations, increased post-harvest sequestration rates and designation of set-aside areas for protection from logging activity | Enhanced carbon sequestration from improved forest management activities | 8-9 | Insufficient data | 0.1-2.1 | Capture: med, yes Storage: med, yes |
|  | Grassland fire management | Avoided emissions from grasslands fires | | Insufficient data | | | |
| Forests and other ecosystems-restore | Reforestation and Afforestation | Carbon sequestration by shifting from non-forest cover to forest cover at 30% tree cover threshold with a region-specific mix of plantation forestry and natural forest regrowth | Carbon sequestration from afforestation and reforestation (forests as defined by FAO) | 8-9 | 0 - 240 | 0.5-10 | Capture: high, yes Storage: high, yes |
|  | Mangrove restoration | Carbon sequestration from restoring mangroves lost since 1996, after excluding those converted to urban land or lost to erosion. | | 8-9 | Insufficient data | 0.5-2.1 | Capture: low, yes Storage: low, yes |
|  | Peatland restoration | Avoided GHG emissions (CO2. CH4 and N2O) from restoration (re-wetting) of degrade peatlands | |  |  |  |  |
| Agricultural  carbon  sequestration | Agroforestry | Carbon sequestration from adding aboveground woody carbon storage in agriculture systems (crop and pasture pixels with <25% tree cover) | | 8-9 | Insufficient data | 0.3-9.4 | Capture: med, yes Storage: med, yes |
|  | Biochar from crop residues | Enhanced carbon sequestration by amending agricultural soils with biochar, which increases the agricultural soil carbon pool by converting rapid-mineralizing carbon (crop residue biomass) to persistent carbon (charcoal) through pyrolysis | | 6-7 | 10 - 345 | 0.3-6.6 | Capture: high, yes  Storage: med, yes |
|  | Soil organic carbon in croplands | Enhanced soil organic carbon sequestration by shifting from current management to no-till management with an input scenario consistent with cover cropping. | | 8-9 | -45 - 100 | 0.6-9.3 | Capture: med, yes Storage: low, yes |
|  | Soil organic carbon in grasslands | Enhanced soil organic carbon sequestration in managed pastures, by shifting from current practices to improved sustainable management with light to moderate grazing pressure and at least one improvement. For rangelands, a shift from current management defined by land degradation to nominally managed | |  |  |  |  |
| BECCS | Bioenergy with Carbon Capture and Storage | Carbon sequestration from electricity generation derived by combusting lignocellulosic crop-based biomass (Miscanthus, switchgrass, short-rotation coppiced trees such as poplar and Eucalyptus) and combined with carbon capture and storage. This excludes the energy substitution effect | | 5-6 | 15 - 400 | 0.5-11 | Capture: high, yes Storage: high, yes |

precision of quantifying the amount of carbon removed (low/med/high/very high) and whether the methodology of MRV exist for the technology (yes/no)[2]. As can be seen from the table, most of the land-based CDR options included in our potential estimates have high TRL (~level 8 to 9) but low/median MRV level compared to BECCS and DACCS.

In addition to the technologies mentioned earlier, there are other options available, such as enhanced weathering, ocean alkalinization, ocean fertilization, coastal wetland management, and more. However, these technologies generally have lower TRL levels (ranging from 1 to 4) and limited MRV capabilities for both capture and storage monitoring. Future research can incorporate the CDR potential of these technologies once their potential is available. By incorporating these alternative options, countries can address their CDR needs more effectively and strive for a more balanced approach to net zero.

## Land-based CDR portfolio for countries

Since BECCS is at the centre of CDR discussion, we include a separate comparison between equitable CDR liability and crop-based BECCS potential^[4]^, and contrast this to removal potential from other land-based solutions^[1]^ and carbon storage potential^[5]^ (**Supplementary Fig.1**). Cumulative CDR liabilities from 2020 to 2050 for the ten major economies are similar under six equity principles, with some variation across IAM-based pathways (as indicated by the error bars).

Compared with the highest CDR liability among six equity principles, domestic land-based CDR potential is tight for six of the ten economies, especially the UK and Japan. The land-based removal potential of the UK can meet only half of its removal liability allocated using the capacity principle and a quarter of its removal liability using the responsibility principle. The UK could resort to technical CDR given the spare geographical storage capacity it has. However, other factors, such as energy demand or high costs, might still hinder technical CDR deployment in practice. The land-based potential in Japan can meet its removal liability only based on the equal per capita principle, while its liability under other equity principles is approximately 1.5 times what the land-based solution can provide. Given its total geophysical storage capacity and land-based removal capacity, Japan still has a chance to meet its national removal liability if it fully deploys all possible technical approaches to fill its geophysical storage sites. In addition to the two countries (Japan and UK) mentioned above the EU-27, India and the US have lower land-based removal potential compared to their liabilities under one or two equity principles. China's CDR potential can meet the average equitable allocation but is insufficient for the maximum allocation results under the six equity principles. For these six economies, realizing the upper bound of their CDR liabilities would require additional, technical (non-land based) solutions to remove carbon.





Supplementary Fig.1 Comparison between CDR liability and capacity for ten major economies (potential estimates from Roe et al.[1], storage estimates from Wei et al.[5]).

National CDR strategies should be tailored given countries' biophysical and geophysical endowments. **Supplementary Fig.2** shows the removal portfolio for ten major economies, including nine land-based solutions and BECCS with a cost-effective hypothesis of a carbon price of $100/tCO_2_. Agriculture removal potential dominates in countries such as China, India, and Australia, where the national biophysical endowment allows enormous potential for amending agricultural soils with biochar and agroforestry. Improved forest management also has great potential to enhance carbon sequestration, which can be the dominant removal solution for Japan. A/R is receiving widespread recognition as a dominant CDR approach, but the related removal share is not much compared to other agricultural solutions except in Canada.

Again, from the portfolio, countries' removal potentials clearly vary, and the removal policy needs to be tailored given their biophysical endowment. The potential is feasible only if countries carefully plan and implement the portfolio with the right deployment strategies (e.g., scale, method, complementarity with other measures and sectors) and geographical context (current biome dynamics, climate, food system, land ownership, etc.)[1].


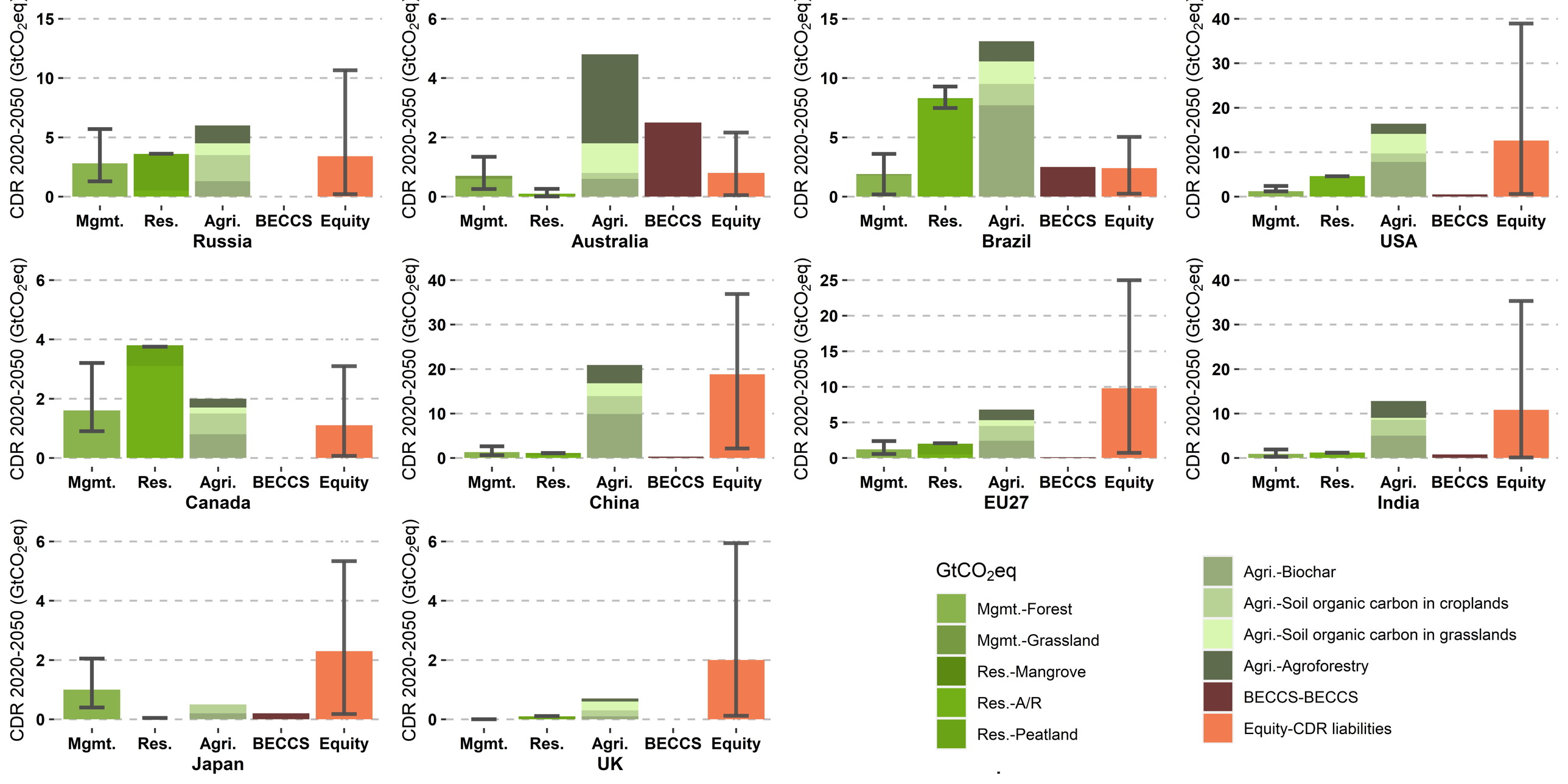


Supplementary Fig.2 Land-based removal portfolio for ten major economies compared with average CDR liability allocated from six equity principles. The nine land-based removal solutions may have double counting issue when summing up with the BECCS potential (potential data adopted from Roe et al.,[1]).

## Public Research, Development and Demonstration

In order to address the apparent global disparity between CDR liability and capacity revealed in our study, it will be necessary to prioritize earlier efforts to reduce emissions and to increase research, development, and demonstration (RD&D) of CDR technologies. Funding from the public sector and demonstration projects can provide valuable insight into a country's CDR capabilities in the future. Therefore, this section presents the latest information obtained from the CDR report [2] along with the authors' own modifications. Supplementary Table 2 highlights funding details, indicating that China takes the lead in land-based CDR investments, specifically focusing on forestry and grasslands. The United States and Germany also invest in land-based CDR options, but these are not their primary areas of focus. In the US, both Congress and the Department of Energy display sustained interest in DACCS. Ocean-based CDR research receives dedicated EU and German Federal Ministry funding. The UK's budget encompasses a wide range of CDR options and also includes methane capture and usage from livestock. This funding information can offer valuable insights to business units regarding the direction of national CDR development, enabling informed investment decisions in CDR technologies.

Supplementary Table 2 Public Research (adopt from [2] with authors' modification)

| Country | CDR option | Funding  (million) | Year | Funding body |
| --- | --- | --- | --- | --- |
| Australia | DACCS | $2.5 m | 2021 | Australian government |
| China | Forestry and grasslands | $21000 m | 2018 | Central government |
| European Union | BECCS | $180 m | 2018 | EU Innovation Fund |
|  | Ocean-based CDR methods (OceanNETs) | $7.2 m | 2018 |  |
| Germany | Ocean-based CDR methods and marine CO2 storage methods (CDRmare) | $26 m | 2021 | German Federal Ministry of Education and Research |
|  | Land-based CDR methods (CDRterra): projects on DACCS, biochar, enhanced rock weathering, BECCS, and A/R | $21 m | 2021 |  |
| United Kingdom | Greenhouse Gas Removal (GGR) | $9.7 m | 2017-2022 | UK Research and Innovation (UKRI) Natural Environment Research Council |
|  | Greenhouse Gas Removal (GGR): BECCS, DACCS, Enhanced weathering, Peatland management, Methane captures and removal etc. | $71.7 m | 2021-2025 | Department for Business, Energy & Industrial Strategy (BEIS) Small Business Research Initiative (SBRI) |
|  | Greenhouse Gas Removal Demonstrators Programme (GGR-D) | $34 m | 2021 | UK Research and Innovation (UKRI) Strategic Priorities Fund (SPF) |
| United States | CDR (37.5% funding goes to DACCS) | $40 m | 2020 | US Congress |
|  | CDR (35% funding goes to DACCS) | $63 m | 2021 |  |
|  | CDR (65% funding goes to DACCS) | $104 m | 2022 |  |
|  | DACCS | $18 m | 2021 | Department of Energy's (DOE), Office of Fossil Energy and Carbon Management (FECM) |
|  | DACCS and ocean-based CDR | $30 m | 2022 |  |
|  | DACCS | $3700 m | 2022 | Department of Energy's (DOE) |
|  | Land-based CDR | $49 m | since 2010 | Department of Energy's (DOE) and the US Department of Agriculture |

The information presented in **Supplementary Table 3** highlights the demonstration projects that were operational in 2022. The majority of these projects are located in the United States, with a few in Europe and two in Canada. emerges as the most widely employed CDR technology among the demonstration projects, demonstrating its popularity and effectiveness. Notably, the largest demonstration project, based in Illinois, stands out for its significant removal potential, capable of removing 1 million metric tons of CO_2_ per year. Overall, the table provides an overview of various operational CDR projects worldwide, emphasizing the prevalence of BECCS and showcasing notable examples of promising CDR efforts.

**Supplementary Table 3** CDR Projects in operation (adopt from [2] with authors' modification)

| Name of the Project | Gross Removals (tCO2/year) | Country |
| --- | --- | --- |
| Archers Daniel Midlands Illinois Industrial Sources | 1000000 | USA |
| Occidental Petroleum Group Oxy-White Energy Biofuel-Production | 350000 | USA |
| Kansas Arkalon Bioethanol | 230000 | USA |
| Bonanza Bioenergy CCUS EOR | 100000 | USA |
| Husky Energy Lashburn and Tangleflags CO_2_ Injection in Heavy Oil Reservoirs Project | 80000 | Canada |
| Biorecro Supporting Bio-Energy with Carbon Capture and Storage | 50000 | Sweden |
| Peel Nre, Bioenergy Infrastructure Group In BECCS | 7300 | UK |
| Climeworks Direct Air Capture and Mineralization | 4000 | Switzerland |
| Ocean-Based Climate Solutions Wave-Powered Upwelling/Downwelling for Carbon Sequestration | 2900 | USA |
| Charm Industrial Geological Sequestration of Bio-Oil | 2000 | USA |
| Enhanced Weathering of Basalt Rock as a Method of Atmospheric CO_2_ Removal | 2000 | UK |
| Climate Foundation Growing Seaweed via Marine Permaculture and Sinking It into the Deep Ocean | 1245 | USA |
| MechanicalTrees Direct Air Capture | 1000 | USA |
| Direct Air Capture with geologic storage of CO_2_ | 1000 | USA |
| Eion Enhanced Weathering | 1000 | USA |
| CO_2_-Zero Surface Olivine Weathering | 500 | UK |
| Rockfarm Carbon Removal Walls to Protect Arable Land and Clean the Atmosphere | 378 | USA |
| Carbon Engineering Direct Air Capture of CO_2_ | 365 | Canada |
| Seachange Mineralization Reactors for CO_2_ Removal with Hydrogen Co-Production | 365 | USA |
| Greensand CO_2_ Removal with Olivine | 300 | Netherlands |
| Neustark recycled concrete | 250 | Switzerland |
| Project Vesta Accelerating Olivine Weathering with Wave Power for CO_2_ Removal | 200 | USA |
| Cambridge Carbon Capture Ltd Direct Air CO_2_ Capture and Mineralisation | 100 | UK |
| C-Sink Sinking Biomass for Deepwater Storage | 100 | USA |
| Green Minerals | 99 | Netherlands |

# 2 GHG emission trajectory

The possibility of keeping the increase in the average global temperature within 1.5°C is ultimately dictated by global GHG emissions and the CDR amount. In our analysis, country-level GHG emissions are also essential for determining countries' 'responsibility', as reflected by their national cumulative GHG emissions. However, providing this emission trajectory can be difficult. While the SSPs are generated using integrated assessment modelling, the results are limited in terms of a regional resolution, which means that they are not readily available for country-specific equity analysis. Various equity principles can allocate global emissions to countries with multiple interpretations of equity, capacity, and responsibility. The staged approach, which combines several equity principles for net emission allocations, is popularly discussed. The proxy for different stages can be GDP per capita or carbon intensity, and countries at their early development stage can adopt liabilities under responsibility principles. The liability must shift to alternative equitable principles if countries' GDP per capita or carbon intensity reaches a certain level. However, how to set this level is controversial, which raises more questions.

Therefore, this analysis adopts country-level SSP data, downscaling by carbon intensity convergence, as a proxy for dynamic responsibility allocation and use it to calculate the CDR ratio to residual emissions. Convergence downscaling assumes that a given variable converges among countries within a given region. The convergence assumption makes sense only for variables independent of a country's size, e.g., emission intensity and GDP per capita. In Gütschow et al.[6], global data are downscaled to the country level using three alternative techniques. In addition to the emission intensity convergence data we used, other methods assume that all countries in a region will have the same emission growth rates defined by the regional or constant emission shares as a reference case independent of the socioeconomic scenario. While there is still a lack of theoretical evidence of a constant emission growth rate within regions and countries' emission shares are barely constant, we follow the authors' recommendation to use exponential convergence downscaling data instead. The emission trajectory downscaled from global data is shown in **Supplementary Fig.4**, and we further compare it with the NDCs and net-zero commitment for reference.

The emission trajectory downscaled from global data is cost-effective, assuming that developing countries are receiving enough financial support to reduce their emissions at low mitigation costs. Country-level emission trajectories are used as a proxy to allocate CDR liabilities under the responsibility principle. The emissions in 2030 are mostly in accordance with the national commitments under the Paris Agreement. According to the national pledges for 2030, the NDCs of India, Russia, and Brazil are higher than the 1.5°C scenario suggests, indicating a need for further emission reduction or CDR in addition to the CDR quotas that we allocate. While China has pledged to have a CO_2_ emissions peak before 2030 and to achieve carbon neutrality before 2060, during COP26, India also pledged to achieve net-zero carbon emissions by 2070. These important commitments expand the possibility of maintaining the global temperature increase within 1.5°C and suggest an opportunity for CDR efforts within these two countries to achieve net-zero.


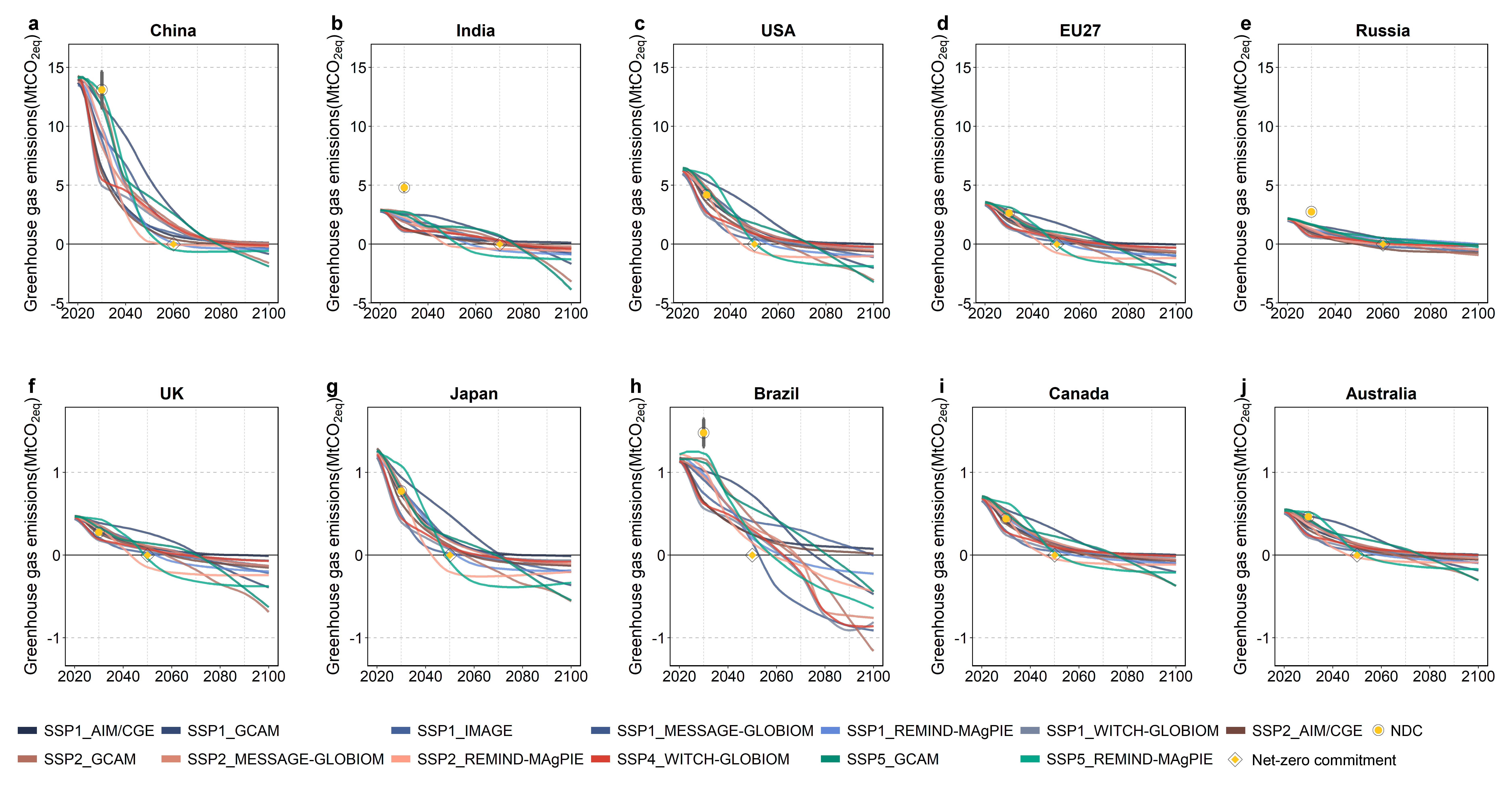


Supplementary Fig.4 National GHG emissions of ten major economies under different SSPs consistent with the 1.5°C goal (plot by author, data from Huppmann et al.,[7] and Meinshausen et al.[8]).

# 3 Potential based CDR allocation

There are 26 countries unable to fulfil their CDR liability domestically, according to their land-based CDR potential estimates. Given that the currently equitable allocation is not feasible domestically to meet the 1.5°C goals, we proposed an alternative allocation method based on national CDR capacity. This alternative allocation assumes countries can cooperate over CDR projects and provide sufficient financial support from developed countries to developing countries. Countries with higher land-based CDR potential shall conduct more CDR to fulfil the global 1.5°C goals.

National CDR capability to deploy CDR is determined by the least of its indigenous removal and storage potentials. Given that the technical CDR has not yet been deployed at large scales and national potential for technical CDR is still unclear, the national capability to remove carbon only considers the land-based CDR methods. While land-based CDR stores carbon in natural systems, and geographical storage potential only constrains the technical CDR deployment, national CDR capability here equals land-based CDR potential.

The potential-based allocation suggests developing countries that are rich in the biophysical CDR endowment to increase effort in CDR endowment (**Supplementary Fig.5**). The allocation relies on the IAM's assumption of harmonized international policies and a fully functional emission trading system to provide financial support for CDR implementation. Compared with equitable allocation results, countries like Japan and the UK can deploy less CDR domestically while transferring their financial support to developing countries like Brazil, which have higher land-based CDR potential, to pursue equity. Note that the allocation is only aiming to avoid the mismatch situation in CDR given the current CDR potential estimates. With promising DAC projects in Iceland and future technical innovation, technical CDR potential can be considered and added to CDR capability accounting.


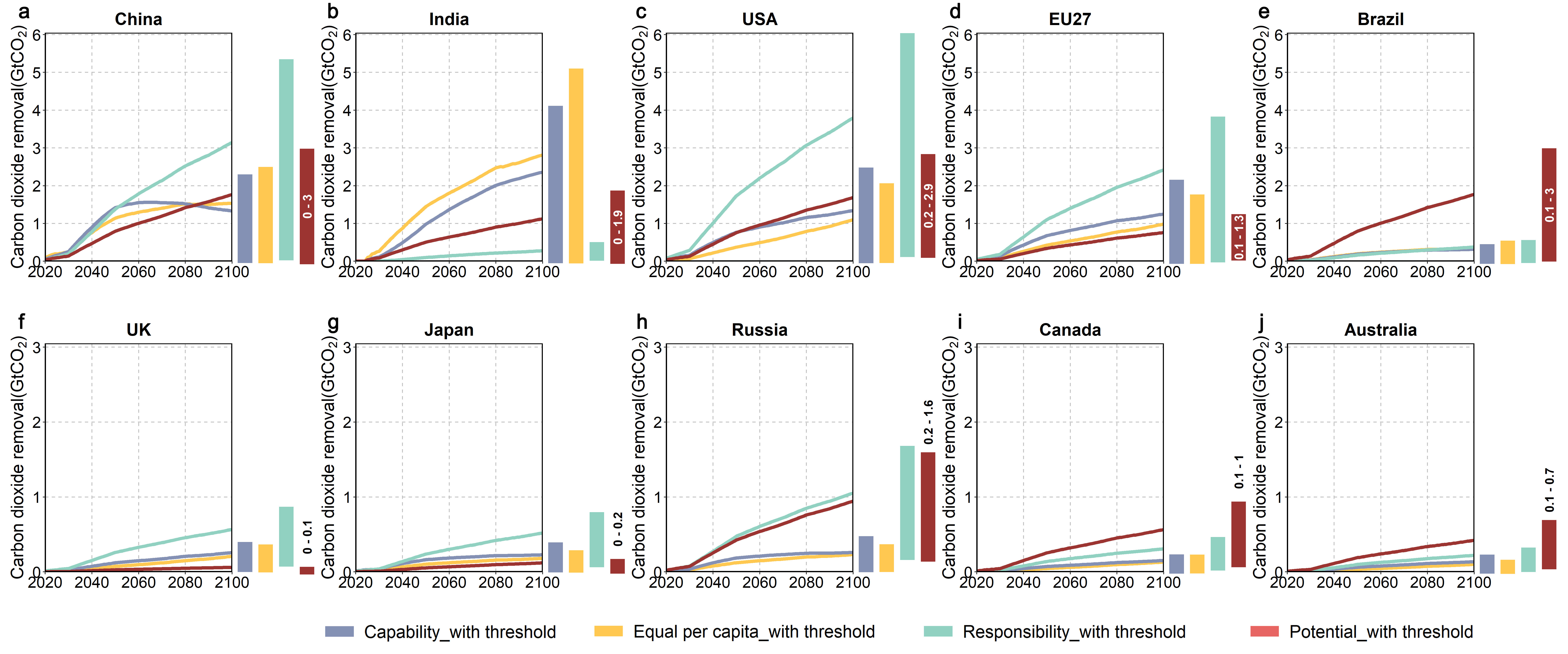


**Supplementary Fig.5** Potential-based allocation results and the equitable allocation for ten parties.

Supplementary References

1. Roe S, Streck C, Beach R, et al. Land-based measures to mitigate climate change: Potential and feasibility by country. Global Change Biology 2021; **27**:6025–6058.
2. Smith SM, Geden O, Nemet G, et al. The State of Carbon Dioxide Removal - 1st Edition. 2023.
3. Griscom BW, Adams J, Ellis PW, et al. Natural climate solutions. Proc Natl Acad Sci 2017; **114**:11645-11650.
4. Hanssen SV, Daioglou V, Steinmann ZJN, et al. The climate change mitigation potential of bioenergy with carbon capture and storage. Nat Clim Chang 2020; **10**:1023–1029.
5. Wei YM, Kang JN, Liu LC, et al. A proposed global layout of carbon capture and storage in line with a 2 °C climate target. Nature Climate Change 2021; **11**:112–118.
6. Gütschow J, Jeffery ML, Günther A, et al. Country-resolved combined emission and socio-economic pathways based on the Representative Concentration Pathway (RCP) and Shared Socio-Economic Pathway (SSP) scenarios. Earth System Science Data 2021; **13**:1005-1040.
7. Huppmann D, Kriegler E, Krey V, et al. (eds). IAMC 1.5°C Scenario Explorer and Data hosted by IIASA. Integrated Assessment Modeling Consortium & International Institute for Applied Systems Analysis, 2018.
8. Meinshausen M, Lewis J, McGlade C, et al. Realization of Paris Agreement pledges may limit warming just below 2 °C. Nature 2022; **604**:304-309.
